# Current status of surviving patients with arginase 1 deficiency in Japan

**DOI:** 10.1016/j.ymgmr.2021.100805

**Published:** 2021-10-01

**Authors:** Jun Kido, Shirou Matsumoto, Eiko Takeshita, Chiemi Hayasaka, Keitaro Yamada, Jiro Kagawa, Yoko Nakajima, Tetsuya Ito, Hiroyuki Iijima, Fumio Endo, Kimitoshi Nakamura

**Affiliations:** a Department of Pediatrics, Faculty of Life Sciences, Kumamoto University, Kumamoto City, Kumamoto, Japan; b Department of Pediatrics, Yanagawa Institute for Developmental Disabilities, International University of Health and Welfare, Yanagawa City, Fukuoka, Japan; c Haruhi Children's Clinic, Kounan City, Aichi, Japan; d Department of Pediatric Neurology, Aichi Developmental Disability Center Central Hospital, Kasugai City, Aichi, Japan; e Department of Pediatrics, Fujieda Municipal General Hospital, Fujieda City, Shizuoka, Japan; f Department of Pediatrics, Fujita Health University School of Medicine, Toyoake City, Aichi, Japan; g Department of General Pediatrics & Interdisciplinary Medicine, National Center for Child Health and Development, Tokyo, Japan

**Keywords:** Arginase 1 deficiency, Cholestasis, Epilepsy, Hyperargininemia, Liver transplantation, CT, Computed tomography, GAA, Guanidino acetate, HPT, Hepaplastin test, LT, Liver transplant, NBS, Newborn screening, UCD, Urea cycle disorder, WAIS, Wechsler Adult Intelligence Scale, WISC, Wechsler Intelligence Scale for Children

## Abstract

Arginase 1 (ARG1) deficiency is a rare urea cycle disorder (UCD), with an estimated frequency of 1 per 2,200,000 births in Japan. Patients with ARG1 deficiency develop symptoms in late infancy or pre-school age with progressive neurological manifestations and sometimes present with severe hepatic disease. We previously investigated the status of UCDs in Japan; however, only one patient was identified as having ARG1 deficiency. Therefore, we aimed to investigate the current status of patients with ARG1 deficiency in 2018–2021 because almost 10 years have passed since the previous study. We present the disease history, clinical outcome, and treatment of five surviving patients with ARG1 deficiency and discuss the features of ARG1 deficiency in Japan. We found that clinicians often face difficulty in diagnosing ARG1 deficiency at the early stage of onset because of interpatient variability in onset time and clinical manifestations. Blood L-arginine and guanidino compounds were considered to be the major factors causing adverse neurodevelopmental outcomes. Therefore, early detection and intervention of ARG1 deficiency is essential for improved neurodevelopmental outcomes. Liver transplantation has been considered an effective treatment option that can dramatically improve the quality of life of patients, prior to the neurological manifestation of symptoms caused by ARG1 deficiency.

## 1 Introduction

Argininemia or arginase 1 (ARG1) deficiency (MIM number: 207800) is an autosomal recessive disorder caused by a defect in ARG1 (L-arginine-urea-hydrolase; EC 3.5.3.1) in the liver. ARG1 catalyzes the final step of urea synthesis in the urea cycle, namely, the hydrolysis of L-arginine to L-ornithine and urea. Urea is excreted through the kidneys, whereas ornithine is returned to the mitochondria and converted to citrulline via the urea cycle. ARG1 deficiency is a rare urea cycle disorder (UCD), and its frequency is estimated to be 1 per 2,200,000 births in Japan [1]. The UCD Consortium study from Europe and the USA reported that the incidence of ARG1 deficiency was estimated to be approximately 1 per 950,000 births [2], accounting for 3.5% of all UCD patients [3]. Increasing incidences are being recognized in the French-Canadian population owing to a founder effect in Northern Quebec [4] and Portugal [5].

Patients with ARG1 deficiency manifest symptoms in late infancy or pre-school age with progressive loss of psychomotor functions, spastic tetraplegia, hyperactivity of deep tendon reflexes, seizures, and growth retardation [6]. Moreover, some patients with ARG1 deficiency may present with severe hepatic diseases, such as neonatal cholestasis, acute liver failure, liver fibrosis, and/or hepatocellular carcinoma [5], [6], [7].

Clinical features of UCDs other than ARG1 deficiency are generally related to recurrent episodes of hyperammonemia. However, the blood ammonia levels in patients with ARG1 deficiency are likely not as elevated compared to those in patients with other UCDs. Moreover, neurological symptoms, such as a marked degree of spastic diplegia, developed in patients with ARG1 deficiency are not a common feature in patients with other UCDs. These neurological symptoms may be progressive even when the blood ammonia levels are within the normal range. For patients with ARG1 deficiency, hyperammonemia is not the sole factor contributing to neurological damage. Hyperargininemia in ARG1 deficiency is related to elevated L-arginine levels and alterations in L-arginine metabolites. Accumulation of L-arginine activates alternative pathways for L-arginine degradation. Abnormalities in the metabolism of guanidino compounds and nitric oxide (NO) results in adverse neuronal effects [8], [9].

We previously investigated the status of UCDs in Japan; however, only one patient was identified as having ARG1 deficiency [10], [11]. In 2018–2021, we resurveyed the history of diagnosis of ARG1 deficiency in nationwide hospitals, with a bed capacity larger than 300, to investigate the current status of ARG1 deficiency in patients. We identified 7 surviving patients who were definitively diagnosed with ARG1 deficiency. Moreover, we acquired the current status of five patients with ARG1 deficiency.

Herein, we present their present status, including disease history, clinical outcome, and treatment of 5 cases of ARG1 deficiency in Japan and compared their clinical features and outcomes compared to cases reported from other countries and those previously reported in Japan.

## 2 Material and methods

In 2018, we sent a questionnaire to 1009 institutions in Japan contacting doctors who had diagnosed or provided medical care for patients with ARG1 deficiency at their respective Departments of pediatrics, endocrinology and metabolism, neonatology, genetics, and transplant surgery. Each institution that received the questionnaire was a medical center assigned to a local area in Japan with a maximum occupancy of 300 or more beds. Among the 1009 institutions, 731 (72.4%) provided responses to our questionnaire and of these 731 institutions, 6 institutions had reportedly treated 7 patients with ARG1 deficiency.

In 2019–2021, we investigated the clinical outcomes of these 7 patients with ARG1 deficiency and analyzed the clinical manifestations, family history, enzyme activity, blood amino acid levels, and/or DNA analysis in five patients who provided informed consent. Cognitive testing for intellectual disability was performed by a pediatrician or child psychiatrist experienced in treating patients with ARG1 deficiency. A severe intellectual disability state was diagnosed when the patient's IQ was <35 according to standardized tests such as the Wechsler Intelligence Scale for Children (WISC) and the Wechsler Adult Intelligence Scale (WAIS). For patients with ARG1 deficiency, these tests were performed at a single center.

This study was approved by the Ethics Committee of the Faculty of Life Sciences, Kumamoto University. Informed consent was obtained from the patients' parents.

## 3 Results

**Fig. 1:**
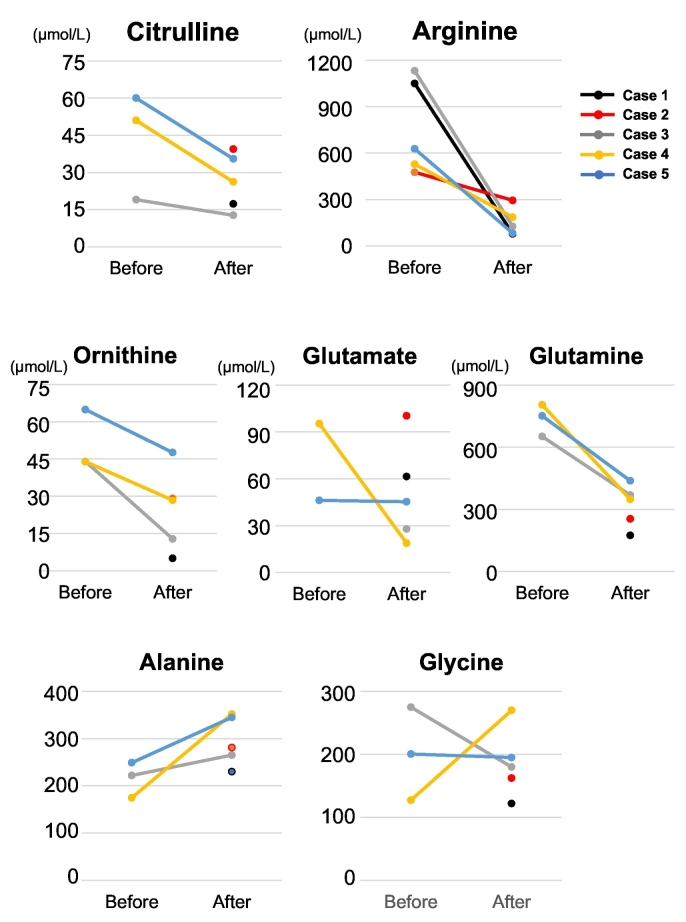
Blood amino acids levels in patients with ARG1 deficiency before and after treatment. The blood arginine levels (Arg: control range: 53.6–133.6 μmol/L) in all cases, dramatically decreased following treatment. The blood citrulline (control range: 17.1–42.6 μmol/L), ornithine (control range: 31.3–104.7 μmol/L), glutamate (control range: 12.6–62.5μmol/L), glutamine (control range: 422.1–703.8 μmol/L) levels decreased and alanine (control range: 422.1–703.8 μmol/L) levels increased in cases 3 to 5 due to the lowered blood arginine levels after treatment.

We present 5 case studies of patients with ARG1 deficiency in Japan and summarize their clinical status in July 2021 (Table 1). Moreover, we report the blood amino acid levels before and after treatment (Fig. 1).

**Table 1:** Clinical summary of 5 patients with ARG1 deficiency in Japan.

	Case 1	Case 2	Case 3	Case 4	Case 5
Sex	Female	Male	Male	Male	Male
Age (years)	45	37	28	25	14
Height (SD)	130 cm (<−3.0SD)	125 cm (<−3.0SD)	144 cm (<−3.0SD)	150 cm (<−3.0SD)	170 cm (+0.7SD)
Onset time	3 weeks	9 months	6 months	1 month	1 month
Diagnosis time	4 years	1 years	9 years	3 months	2 months
Maximum NH_3_ levels (μmol/L)	565	300	262	1970	177
Liver transplantation	(−)	(−)	(−)	(−)	(+)
Enzyme assay	Yes	Yes	Yes	Yes	Yes
DNA analysis	c.78delA (p.Gly27Alafs*5), c.263-266delAGAA (p.Lys88Argfs*45)	c.365G > A (p.Trp122*), c.703G > C (p.Gly235Arg)	ND	ND	c.365G > A (p.Trp122*)*,* c.820G > A (p.Asp274Asn)
Growth disorder	(+)	(+)	(+)	(+)	(−)
Anorexia	(−)	(−)	(+)	(−)	(−)
Nausea or vomiting	(+)	(−)	(+)	(+)	(−)
Diarrhea	(+)	(−)	(−)	(−)	(−)
Elevated transaminases (>100 U/L)	(+)	(−)	(+)	(+)	(+)
Coagulopathy	(−)	(−)	(−)	(−)	(+)
Spastic diplegia	(+)	(+)	(+)	(+)	(−)
Convulsion	(+)	(+)	(+)	(+)	(−)
Intellectual disability	(+) Severe	(+) Severe	(+) Severe	(+) Severe	(−)
Cerebral palsy	(+)	(+)	(+)	(+)	(−)
Hypertonia	(+)	(+)	(+)	(−)	(−)
Muscle atrophy	(+)	(+)	(+)	(+)	(−)
Abnormal brain CT or MRI	(+)	(+)	(+)	(+)	(−)
Abnormal EEG	(+)	(+)	(+)	(+)	(−)

ND: not done.

### 3.1 Case 1 (45 years, female)

The patient presented to hospital because of severe hyperammonemia at the age of 4 years. She was born at 42 weeks' gestation, weighing 3300 g, by normal delivery. She was born to non-consanguineous parents. At the age of 18 days, she frequently vomited after feeding on breast milk. She presented with febrile convulsions at the age of 23 days. The tonic-clonic convulsions persisted, and the Moro reflex was defective. Her convulsions were difficult to control despite receiving phenobarbital and phenytoin treatment. Brain computed tomography (CT) imaging at age of 12 months demonstrated bilaterally dilated cerebral ventricles and subdural fluid storage. The frequency of convulsions increased from the age of 18 months, and she presented with poor weight gain from the age of 3 years. At the age of 4 years and 2 months, she developed pneumonia, and her blood aspartate aminotransferase (AST, 720 IU/L), alanine aminotransferase (ALT, 335 IU/L), lactate dehydrogenase (LDH, 974 IU/L), ammonia (565 μmol/L), and arginine (1049 μmol/L) levels were dramatically elevated. She presented a decorticate posture, with enhanced deep tendon reflexes and muscle tension in her extremities. She could not roll over or speak, and her head control was poor. Her erythrocytes exhibited diminished arginase activity, with less than 1% of control activity. Genetic analysis identified compound heterozygous mutations (c.78delA [p.Gly27Alafs*5] and c.263-266delAGAA [p.Lys88Argfs*45]). Electroencephalography (EEG) results demonstrated spikes in the temporal and frontal lobes. Brain CT showed significant enlargement of the bilateral, cerebral, and third ventricles. At 45 years of age, she presented with increased deep tendon reflexes, scissoring, spastic quadriplegia, sardonic laugh seizures, and periodic vomiting, and was bedridden.

### 3.2 Case 2 (37 years, male)

The patient presented to hospital because of the development of impaired consciousness with a common cold at the age of 9 months. He was born at 39 weeks of gestation weighing 2972 g by normal delivery. He exhibited a fixed neck at the age of 4 months and presented otherwise normal development up to 8 months. At the age of 9 months, he experienced impaired consciousness with viral infection and was started on an arginine-free formula owing to hyperargininemia (644 μmol/L). Subsequently, the patient frequently presented with seizures and received anti-seizure drugs. At the age of 1 year and 3 months, he showed evidence of brain atrophy and subdural hematoma on the brain CT. He was frequently hospitalized because of pneumonia as an infant and at school age. He underwent tracheostomy because of an unstable respiratory state at the age of 20 years, gastrostomy at age 21 years, and laryngotracheal separation at the age of 22 years. He presented no arginase activity or compound heterozygosity of c. 365G > A (p. Trp122*) and c. 703G > C (p.Gly235Arg) mutations. At the age of 37, he currently lives a bedridden life.

### 3.3 Case 3 (28 years, male)

He developed infantile epilepsy at the age of 6 months. He presented with liver dysfunction after receiving sodium valproate; however, his infantile epilepsy was controlled by combination treatment with clonazepam and vitamin B6. He presented with mild spasticity in both lower limbs and moderate growth delay at the age of 1 year and 5 months. By the age of 3 years, he developed partial epilepsy with flexed upper limbs and extended lower limbs; however, the epilepsy disappeared after carbamazepine treatment. At the age of 7 years, he presented with atypical absence seizures. Additional ethosuximide treatment improved his absence attacks. At the age of 9 years, he could not eat, became somnolent, and was hospitalized for further investigation. His blood ammonia (268 μmol/L) and arginine (476 μmol/L) levels were significantly elevated. Arginase activity in erythrocytes was not detectable. He was diagnosed with ARG1 deficiency and was managed with a restricted protein diet, special formulas devoid of arginine, and sodium benzoate. After receiving these treatments, his blood arginine levels were controlled at 80–140 μmol/L. His epileptic attacks decreased, and his EEG was near normal. At the age of 19 years, the brain magnetic resonance imaging (MRI) demonstrated bilateral subcortical white matter atrophies from temporal to occipital lobes and cerebellar atrophy.

At the age of 28 years, he had developed severe intellectual disability and was still unable to walk. His seizures persisted despite receiving medical treatment. He currently lives at home with his family and receives social services daily.

### 3.4 Case 4 (25 years, male)

The patient was admitted after having developed an abnormal respiratory condition at the age of 1.5 months. He was born at term weighing 3544 g by normal delivery, with APGAR scores of 9 at 1 min and 9 at 5 min after birth. He had non-consanguineous parents. His brother died of accidental ingestion at the age of 8 months following failure to thrive and concurrent developmental delay with an unknown cause. He was exclusively breast-fed, but had poor overall health and presented with poor suckling at 43 days after birth, with continuous hiccups synchronized with breathing and lethargy. The laboratory tests showed a compensated respiratory alkalosis (pH: 7.47, pCO_2_: 20.1 mmHg, HCO_3_^−^; 14.4 mmol/L, base excess [BE): −8.6 mmol/L), severe hyperammonemia (1970 μmol/L), and slightly elevated ALT levels (71 IU/L). He underwent peritoneal dialysis, and his blood ammonia levels decreased to 102 μmol/L after 12 h. The prolonged hiccups subsided, and the patient's appearance improved. Blood amino acid analysis revealed that arginine (1131 μmol/L) was markedly elevated with increased urinary excretion of arginine (6964 μmol/L), although glutamate (95 μmol/L) and glutamine (805 μmol/L) remained mildly elevated. Red blood cells presented no arginase activity (<1 μmol/h/g hemoglobin), and the patient was diagnosed with ARG1 deficiency. CT showed slight cerebral atrophy with enlargement of the sulci and ventricles. EEG revealed occasional, diffuse, and slow-wave bursts. He developed mild-to-moderate motor delay one year after onset (head control: 6 months, roll over: 8 months, and sitting without support: 12 months) being treated with a protein-restricted diet with arginine-free formula.

At the age of 25 years, he received sodium butyrate, sodium benzoate, l-carnitine, and arginine-free formula treatment. He lives at home with his family and goes to a training center for regular rehabilitation.

### 3.5 Case 5 (14 years)

A 1 month old, male infant was hospitalized because of an impaired hepaplastin test (HPT: 26%, control: 70–130%), which was a test measuring activity of blood coagulation factor such as II, VII and X. He was born at 36 weeks and 6 days of gestation and weighed 3160 g. He underwent light therapy 5 days after birth because of hyperbilirubinemia. He was treated with vitamin K, but his impaired HPT persisted for one month. Moreover, he developed mild cholestatic liver disease (AST: 72 IU/L, ALT: 45 IU/L, total bilirubin: 4.5 mg/dL, and direct bilirubin: 1.6 mg/dL) with hyperammonemia (177 μmol/L) and hyperargininemia (1402 μmol/L). He was diagnosed with suspected ARG1 deficiency and received medical treatment, including protein-free formulas, sodium benzoate, and carnitine. The patient's blood ammonia (35 μmol/L) and arginine (129 μmol/L) levels and coagulopathy (HPT: 79%) improved following medical treatment. Arginase activity in erythrocytes was not detectable, and genetic analysis identified the presence of compound heterozygous mutations (c.365G > A (p.Trp122*) and c.820G > A (p.Asp274Asn)) in the *ARG1 gene.* The patient underwent a living donor liver transplant (LT) at the age of 1 year and 5 months. Therefore, he had not experienced hyperammonemia, and his abnormal amino acid metabolites were corrected after LT without additional medical treatment.

At the age of 14 years, he had normal neurodevelopment and lived a stable life with a normal school routine and no medical problems.

## 4 Discussion

We present 5 case studies of patients with ARG1 deficiency in Japan. The patients developed various symptoms that could be divided into neurological and/or liver-type diseases, with varying blood ammonia levels. Cases 1–4 developed a mentally and physically handicapped state, and case 5 did not acquire any disability. All patients in this study, except for case 5 who received LT, presented short stature. No patient presented with HCC.

Many clinicians have significant difficulty in diagnosing ARG1 deficiency in its early stages. However, because all patients with ARG1 deficiency in Japan are diagnosed with hyperargininemia based on the blood amino acid analysis, it is very important to perform this test if patients present symptoms such as growth impairment, seizure, paralysis, liver disorder, and cholestasis.

Patients with ARG1 deficiency are likely to exhibit lower blood ammonia levels compared to patients with other UCDs. However, the blood ammonia levels in our cases 1 and 4 at the time of onset were significantly high, and the blood ammonia levels in cases 2, 3, and 5 were moderately high, suggesting that the blood ammonia levels at the onset time could not distinguish patients with ARG1 deficiency from patients with other UCDs.

In Japan, acylcarnitine and some amino acids are analyzed in all newborns by tandem mass spectrometry (MS/MS) using dried blood spots. However, not all prefectures in Japan target arginine in newborn screening (NBS) by MS/MS. None of our five patients underwent NBS with MS/MS. If arginine analysis had been performed using NBS with MS/MS, hyperargininemia may have been detected prior to the onset of neurological manifestations [12] and the long-term outcome in patients with ARG1 deficiency may have been improved through early intervention.

A prior report showed that patients with ARG1 deficiency developed neonatal cholestasis, such as in case 5, although it is a rare occurrence [5], [6]. The underlying mechanism in neonatal cholestasis in ARG1 deficiency remains unknown. Some patients with ARG1 deficiency who develop neonatal cholestasis require LT owing to liver failure. Moreover, the mechanisms by which ARG1 deficiency results in either liver disorders or neurological disorders remain unknown. Some reports have described cases of ARG1 deficiency presenting with hyperammonemia in the neonatal period or early infancy [7], [13], [14], [15], [16]. Some of these patients underwent LT, and liver injury levels and neurodevelopmental outcomes differed in each case. Therefore, it is uncertain whether the neonatal onset type was similar to more severe types of ARG1 deficiency.

Arginine and glycine are metabolized into guanidino acetate (GAA) and ornithine by L-arginine-glycine amidinotransferase [17]. Increased blood GAA is considered to contribute to neuropathology in ARG1 deficiency [18] because it induces oxidative stress in the brain [19]. Moreover, other guanidino compounds (other than GAA) have adverse effects on the brain [20], [21].

Arginine also increases oxidative stress by enhancing lipid peroxidation and alleviating antioxidant capacity [22]. Excess arginine administration into the cerebroventricular system increased levels of thiobarbituric acid-reactive substances, which are oxidative stress markers, and compromise both total radical-trapping antioxidant parameter capacity and energy metabolism in the hippocampus [23]. Moreover, arginine reinforces nitric oxide (NO) synthesis [24] and leads to increased synthesis of peroxynitrite from NO and superoxide, which in turn induce oxidative stress [25]. Therefore, elevated blood levels of both arginine and its metabolites have significant adverse effects on the brain.

Many different pathogenic mutations in ARG1 have been detected, and their clinical outcomes have been reported [26], [27], [28], [29], [30], [31]. Our institution first reported patients with ARG1 deficiency in Japan over 20 years ago [26]. Neurodevelopmental outcomes in patients with ARG1 deficiency at that time were extremely poor, even in patients with late-onset ARG1 deficiency. Uchino et al. [26] considered that lower blood arginine levels in patients with ARG1 deficiency were linked to the degree of improvement in neurological factors and that early diagnosis and treatment would achieve greater benefits. Conversely, Huemer et al. [30] hypothesized that the clinical manifestations in patients with ARG1 deficiency did not correlate with protein intake or blood arginine, asymmetric dimethylarginine, and nitrate levels. Therefore, early intervention is considered most likely to preserve motor and cognitive function in patients with ARG1 deficiency [32], [33] although this phenomenon could not be confirmed in all cases [34].

Only case 5 [35] received LT before developing neurological manifestations and had therefore achieved a stable school life with normal intelligence. Leonard et al. [36] described a patient with ARG1 deficiency subjected to complete metabolic correction and achieved improved quality of life with a normalization of diet and a decreased frequency of hospital admissions after receiving LT. Moreover, Silva et al. [37], [38] suggested that LT prevented progressive neurological impairment in patients with ARG1 deficiency. Angarita et al. [39] demonstrated that the metabolic abnormalities developed in mice with ARG1 deficiency could be mitigated or markedly improved with transplantation of normal human hepatocytes. Moreover, there was no significant differences between hepatocyte-transplanted mice without endogenous hepatic arginase activity and age-matched wild-type arginase-positive mice on neurological assessment. Thus, early LT before neurological manifestations may improve long-term neurological outcomes as well as metabolic conditions in patients with ARG1 deficiency.

Interventions for ARG1 deficiency include ammonia scavengers such as sodium benzoate and sodium phenylbutyrate, arginine-free formula, and restriction of protein intake. More recently, pegzilarginase has been reported to be effective as arginase replacement therapy, and is able to reduce blood arginine and glutamic acid levels [40]. Arginase replacement therapy is expected to prevent deterioration of mobility and neurological dysfunction because blood arginine is a major factor with adverse effects on neurodevelopmental outcomes.

This study have some limitations. Only 5 patients were included in this study because ARG1 deficiency is very rare. Moreover, this study is retrospective cohort study. Their clinical manifestations and treatment, which was different on each patients, makes analysis of factors having effect on their clinical outcome difficult.

In conclusion, ARG1 deficiency, a very rare disease and should be detected and treated before the development of neurological manifestations. Early LT may contribute to improving long-term neurological outcomes and general conditions in patients with ARG1 deficiency. Thus, measurement of blood arginine levels through NBS with MS/MS can lead to early diagnosis and treatment of ARG1 deficiency, which may lead to improved neurodevelopmental outcomes.

## Funding

This work was supported in part by a Health and Labor Sciences Research Grant for Research on Rare and Intractable Diseases from the Ministry of Health, Labour and Welfare, Japan (grant number: JPMH20FC1025), a Grant-in-Aid for Practical Research Project for Rare/Intractable Diseases from the Japan Agency for Medical Research and Development (AMED; grant numbers: JP19ek0109276, JP21ek0109482), and a Grant-in-Aid for Scientific Research from the Ministry of Education, Culture, Sports, Science, and Technology, Japan (Japan Society for the Promotion of Science [JSPS] KAKENHI, grant number: JP20K08207).

## Author contributions

J.K and K.N were responsible for the design of the research. J.K, S.M, E.T, C.H, K.Y, J.K, Y.N, T.I, H.I, and F.E contributed to practicing medicine and data collection from patients with ARG1 deficiency. J.K, S.M and K.N checked and analyzed the data. J.K wrote the manuscript. J.K and K.N supervised this study. All authors read and approved the final manuscript for submission. All authors have agreed both to be personally accountable for the author's own contributions and to ensure that questions related to the accuracy or integrity of any part of the work.

## Declaration of Competing Interest

All authors declare that there are no conflicts of interest in relation to the current study.
